# Clinical benefits of adjuvant chemotherapy with carboplatin and gemcitabine in patients with non-small cell lung cancer: a single-center retrospective study

**DOI:** 10.1186/s12957-020-02041-0

**Published:** 2020-10-08

**Authors:** Shinogu Takashima, Kazuhiro Imai, Maiko Atari, Tsubasa Matsuo, Katsutoshi Nakayama, Yusuke Sato, Satoru Motoyama, Hiroyuki Shibata, Kyoko Nomura, Yoshihiro Minamiya

**Affiliations:** 1 Department of Thoracic Surgery, Akita University Graduate School of Medicine, 1-1-1 Hondo, Akita, 010-8543 Japan; 2 Department of Respiratory Medicine, Akita University Graduate School of Medicine, 1-1-1 Hondo, Akita, 010-8543 Japan; 3 Department of Clinical Oncology, Akita University Graduate School of Medicine, 1-1-1 Hondo, Akita, 010-8543 Japan; 4 Department of Health Environmental Science and Public Health, Akita University Graduate School of Medicine, 1-1-1 Hondo, Akita, 010-8543 Japan

**Keywords:** Carboplatin, Gemcitabine, Adjuvant chemotherapy, Lung cancer

## Abstract

**Purpose:**

In cases of non-small cell lung cancer (NSCLC), surgery remains the best option for cure, but surgery is of benefit only when the disease is localized. Although adjuvant chemotherapy reportedly has a significant beneficial effect on survival, the benefit of a carboplatin (CBDCA) regimen is unclear. We therefore investigated the efficacy and tolerability of CBDCA (area under the curve 5) plus gemcitabine (GEM, 1000 mg/m^2^) as adjuvant chemotherapy.

**Methods:**

A total of 82 pStage IB-IIIA NSCLC patients who had undergone complete resection and received adjuvant chemotherapy were analyzed retrospectively. Among them, 65 patients received CBDCA + GEM and 17 received CDDP + VNR. Propensity score analysis generated 17 matched pairs of both groups.

**Results:**

Sixty-five patients received CBDCA + GEM. Their 5-year relapse-free survival (RFS) and overall survival were 47.8% (median, 52.5 months) and 76.9% (median, 90.1 months), respectively. Toxicities, which included neutropenia, nausea/anorexia, fatigue, and vasculitis, were significantly milder than with CDDP + VNR. There were no significant differences in RFS between CBDCA + GEM and CDDP + VNR (*p* = 0.079) after matching for age, performance status, and pStage.

**Conclusion:**

CBDCA + GEM was effective and well tolerated as adjuvant chemotherapy, with a manageable toxicity profile.

## Introduction

Lung cancer is the most common cause of cancer-related death globally. In cases of non-small cell lung cancer (NSCLC), surgery remains the best option for a potential cure. However, surgery is only of benefit to patients with localized disease and no evidence of mediastinal lymph node involvement or distant metastasis. Following surgical resection, patients with stage II–III advanced NSCLC face a high risk of relapse, and the treatment strategy for perioperative advanced NSCLC patients remains unsatisfactory [1, 2].

Adjuvant chemotherapy is now standard treatment for patients with completely resected advanced NSCLC. Randomized controlled trials (RCTs) and meta-analyses have shown that adjuvant chemotherapy improves survival over surgery alone [3–7]. The Lung Adjuvant Cisplatin Evaluation (LACE) meta-analysis reported that cisplatin-based adjuvant chemotherapy had a 5-year survival benefit of 5.4% with an overall hazard ratio for death of 0.89 (95% confidence interval [CI] 0.82–0.96) [5], which represents a significant survival benefit for the patients with stage II or stage III NSCLC [6]. In 2017, the Japanese Guidelines for the Diagnosis and Treatment of Lung Cancer changed and now recommend cisplatin (CDDP)-based adjuvant chemotherapy, most often administered as CDDP + vinorelbine (VNR), with strength of recommendation 1 (high)/evidence quality A (high) based on RCTs and systematic reviews [3, 5–7]. Likewise, the National Comprehensive Cancer Network (NCCN) guideline and Society of Clinical Oncology (ASCO)/Cancer Care Ontario (CCO) clinical practice guideline were updated with the same strength of recommendation [7, 8]. CDDP-based adjuvant chemotherapy was recommended for routine use in patients with stage II or IIIA disease and recommended for consideration in patients with stage IB NSCLCs [7]. This is despite the low CDDP completion rate due to severe toxicities that include nausea, vomiting, and nephrotoxicity.

In its 2018 update, however, the NCCN Guideline added two preoperative and postoperative therapy regimens for patients with comorbidities or those not able to tolerate CDDP: (1) carboplatin (CBDCA)/gemcitabine (GEM) and (2) CBDCA/pemetrexed (non-squamous only) [8]. Although most recent evidence indicates that cisplatin-based regimen is standard as adjuvant chemotherapy for R0 resected pStage II-III NSCLC patients, CBDCA has been found to be an acceptable alternative to cisplatin in doublet chemotherapy [9]. Moreover, evidence suggests the platinum combination agents GEM, paclitaxel (PTX), and docetaxel also have activity as adjuvant chemotherapy agents [5, 10–12].

GEM (pyrimidine antimetabolite, 2',2'-difluorodeoxycytidine) [13] exhibits wide-spectrum of antitumor activity. The combination of platinum with GEM is used clinically for advanced NSCLC [14], and the superiority of GEM-containing regimens in terms of efficacy and toxicity over other cytotoxic chemotherapy regimens has been shown in several studies [15, 16]. Among now-existing cytotoxic regimens for advanced NSCLC, CBDCA with GEM has proven to be one of the best, with definite anticancer efficacy and a manageable toxicity profile without thrombocytopenia [17]. In addition, three phase II trials of CBDCA + GEM have reported that the regimen is effective for disease control and is well tolerated by patients after surgery [18–20]. This regimen is thus one of the adjuvant chemotherapy options suitable for outpatients with completely resected NSCLC.

Although CBDCA + GEM is a feasible and promising regimen for adjuvant chemotherapy with lower levels of toxicity, most of the phase II studies did not compare it with CDDP + VNR, the current standard adjuvant chemotherapy regimen. We hypothesized that given the higher patient tolerance, CDBCA + GEM has the potential to increase the completion rate while decreasing adverse events such as nausea, vomiting, hair loss, febrile neutropenia, and nephrotoxicity. The aim of the present retrospective study was to compare the efficacy and tolerability of the CBDCA + GEM regimen with the standard CDDP + VNR regimen as adjuvant chemotherapy for patients with pathological stage II–III NSCLC who underwent complete surgical resection at a single center.

## Methods

### Study population

This was a single-center, retrospective study of treatment with CBDCA + GEM for patients who had undergone complete surgical resection (R0 resection) for pStage IB–III NSCLC (UICC TNM Classification of Malignant Tumors 8th edition) [21]. This study was performed in accordance with the principles of the Declaration of Helsinki and Good Clinical Practice guidelines. The retrospective study was approved by the institutional review board (IRB) at Akita University Hospital (Approval number/ID 2336), and all data were collected under this IRB Protocol No. 2426, which allows collection of tissue and medical record with consent or waiver of consent when no personalized health information is required, as was the case for this study. We analyzed 82 of 347 pStage IB–III patients who underwent thoracic surgery at Akita University Hospital between January 2009 and August 2019. All 82 patients received adjuvant chemotherapy that did not include tegafur-uracil (UFT). Among that group, 65 patients received CBDCA + GEM and 17 received CDDP + VNR. All patients underwent segmentectomy, lobectomy, or pneumonectomy along with systematic lymph node dissection. The patients’ characteristics are listed in Table 1.

**Table 1:** Patient characteristics. *P<0.05

Patient characteristics	CBDCA + GEM (*n* = 65)	CDDP + VNR (*n* = 17)	*P*
Median age, years (range)	66 (43–79)	59 (33–70)	0.004*
Gender (%)			0.239
Male	51 (78.4)	11 (64.7)	
Female	14 (21.5)	6( 35.2)	
Surgical procedure (%)			0.874
Lobectomy	61 (93.8)	16 (94.1)	
Pneumonectomy	4 (6.1)	1 (5.8)	
ECOG performance status (%)			0.002*
0	36 (55.3)	17 (100)	
1	27 (41.5)	0	
2	2 (3.0)	0	
Pathological stage (%)			0.532
IB	3 (4.6)	0	
IIA	3( 4.6)	1 (5.8)	
IIB	27 (41.5)	4 (23.5)	
IIIA	22 (33.8)	8 (47.0)	
IIIB	10 (15.3)	4 (23.5)	
Histology (%)			0.185
Adenocarcinoma	44 (67.6)	16 (94.1)	
Squamous cell carcinoma	19 (29.2)	1( 5.8)	
Large cell carcinoma	1 (1.5)	0	
Others	1 (1.5)	0	
Lymph nodal status (%)			0.271
N0	15 (23.0)	2 (11.7)	
N1	22 (33.8)	4 (23.5)	
N2	28 (43.0)	11 (47.5)	
Number of course			0.119
1/2/3/4	2/9/5/49	2/0/3/12	
EGFR mutation (%)			0.626
Negative/unknown	53 (81.5)	13 (76.4)	
Positive	12 (18.4)	4 (23.5)	
ALK fusion (%)			0.301
Negative/unknown	64(98.4)	16(94.1)	
Positive	1(1.54)	1(5.8)	

### Adjuvant chemotherapy

Patients receiving CBDCA + GEM were administered GEM at 1000 mg/m^2^ intravenously over 3 h on day 1 and/or day 8. CBDCA at a dose based on an area under the curve (AUC) of 5 was given intravenously over 30 min after GEM on day 1. Patients receiving CDDP + VNR were administered VNR at 25 mg/m^2^ intravenously over 5 min on day 1 and/or day 8). CDDP at 80 mg/m^2^ was given intravenously over 30 min after the VNR on day 1. Chemotherapy was repeated every 4 weeks for up to 4 cycles unless there was evidence of disease progression or unacceptable toxicity. Toxicities were graded using the National Cancer Institute Common Toxicity Criteria (NCI-CTC), version 5.0. Chemotherapy was modified for toxicity and adverse effects as necessary. Patients were then followed up within 2 months after the final chemotherapy cycle and evaluated based on a physical examination, chest X-ray, CT, and laboratory tests. Although the follow-up schedule after surgery varied, it usually entailed a chest CT every 3–6 months and brain MRI, bone scintigraphy, or PET/CT every 6–12 months. After 2 years, the frequency of chest CTs was reduced to once every 6–12 months. If recurrence was suspected, the follow-up schedule was tightened. Radiographic responses were assessed using RECIST ver. 1.1 [22]. Treatment for recurrence was not restricted in the present study.

We established relapse free survival (RFS) as the primary endpoint. It was defined as the time from surgery to recurrence (locoregional and/or distant metastasis or death as a result of any cause). The secondary end-points were to evaluate the overall survival (OS), and clinical safety compared with another regimen.

### Evaluation and statistical analysis

Group data are expressed as means ± standard deviation. Continuous data were compared using unpaired *t* tests or the Wilcoxon/Kruskal-Wallis test, while categorical data were compared using the chi-squared test with continuity correction or Fisher’s exact test when applicable. The Kaplan-Meier method was used to estimate RFS and OS and log-rank test was used to assess the impact of surgery on RFS and OS. *P* values were 2-sided and considered significant if less than 0.05. To control for potential differences in the preoperative characteristics of the patients in the two groups, propensity score matching was used. The propensity scores were generated using logistic regression based on clinically relevant variables such as age, performance status (PS), and pStage, and were considered as possible confounders due to their potential association with the outcome of interest based on clinical knowledge. Patients were matched 1:1 through nearest neighbor matching (caliper width: 0.2) without replacement. To measure covariate balance, we used the standardized difference, whereby an absolute standardized difference above 0.1 represents meaningful imbalance. Statistical analysis was performed using JMP IN 14.2.0 software (SAS Institute, Cary, NC, USA).

## Results

**Fig. 1:**
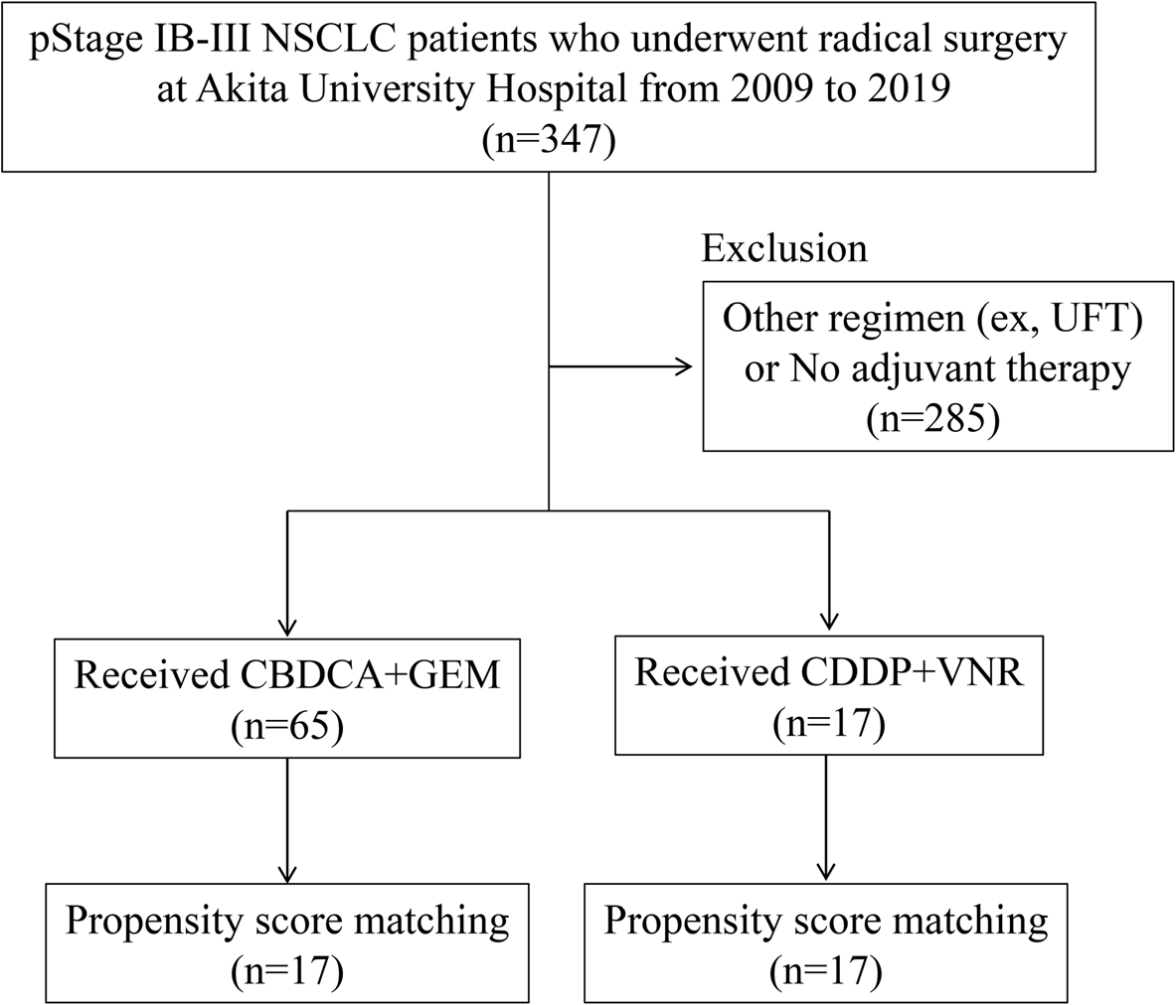
Diagram process used for patient selections. (NSCLC; non-small cell lung cancer)

Between January 2009 and August 2019, 82 pStage IB-IIIA NSCLC patients were deemed eligible for inclusion in this study. A diagram of the selection process is shown in Fig. 1, and the patients’ clinical characteristics are summarized in Table 1. The median follow-up period was 3.19 years (range per trial, 0.48 to 8.28 years). As adjuvant chemotherapy, 65 of these patients received CBDCA + GEM, while the remaining 17 received CDDP + VNR.

**Fig. 2:**
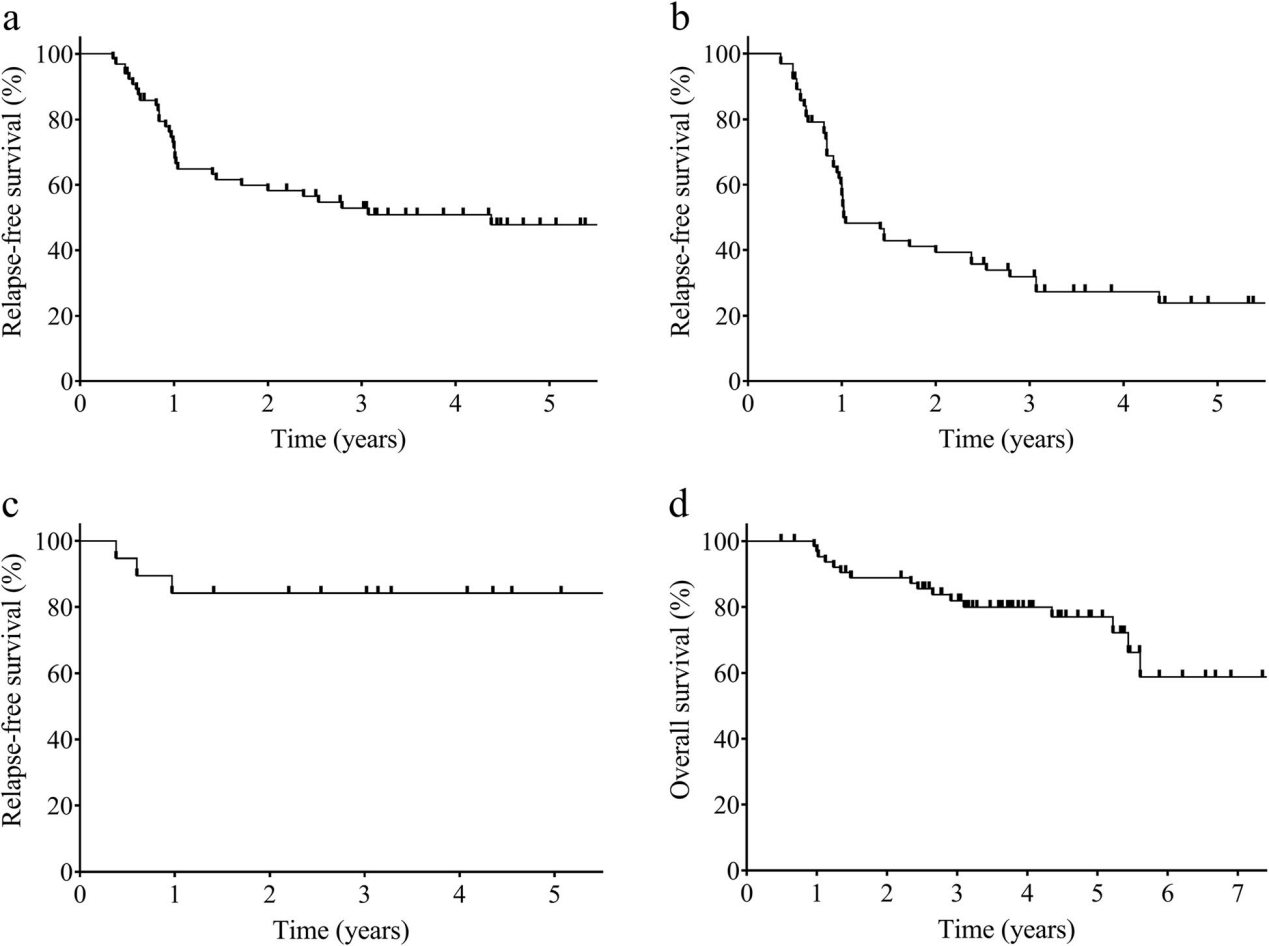
Kaplan-Meier curves of relapse-free survival (**a**) among patients treated with carboplatin plus gemcitabine. Relapse-free survival in patients with adenocarcinoma (**b**) and squamous cell carcinoma (**c**). And overall survival (**d**) among them

The 5-year RFS and OS among patients receiving the CBDCA + GEM regimen were 47.8% and 76.9%, respectively. The median RFS and OS time in the CBDCA + GEM group was 52.5 months (range, 4.2 to 85.6 months, 95% CI 16.9–85.6) and 90.1 months (range, 11.5 to 99.3 months, 95% CI 65.2–99.3) (Fig. 2a, d). Among them, the 5-year RFS in patients with adenocarcinoma and squamous cell carcinoma were 31.5% and 84.2%, respectively, and the median RFS time were 20.6 months (range, 4.2 to 85.6 months, 95% CI 11.8–52.5) and 84.9 months (range, 4.5 to 88.2 months, 95% CI not calculated) (Fig. 2b, c). The 5-year OS in patients with adenocarcinoma and squamous cell carcinoma were 62.6% and 94.4%, respectively, and the median OS time were 98.4 months (range, 5.8 to 99.3 months, 95% CI 62.4–99.3) and 90.0 months (range, 16.9 to 90.0 months, 95% CI 67.3–90.0).

The 5-year RFS and OS among patients receiving the CDDP + VNR regimen were 0% and 100%, respectively. The median RFS time in the CDDP + VNR group was 10.4 months (range, 4.9 to 24.1 months, 95% CI 6.2–11.1) and the median OS time was not reached.

All patients were evaluated for toxicities (Table 2). The observed toxicities were milder in the CBDCA + GEM group, and the patients showed better compliance than in the CDDP + VNR group. Grade 3/4 hematological adverse effects of CBDCA + GEM included neutropenia (15.3%) and thrombocytopenia (4.6%). The non-hematological adverse effects of both regimens consisted mainly of nausea/vomiting. No grade 3/4 non-hematological adverse effects occurred in the CBDCA + GEM group. In addition, the incidences of other mild adverse effects, such as fatigue and vasculitis, were lower in the CBDCA + GEM than CDDP + VNR group.

**Table 2:** Adverse events

NCI-CTC grade (ver.5)	CBDCA + GEM (*n* = 65)			CDDP + VNR (*n* = 17)			
	G1/2 (%)	G3/4 (%)	total	G1/G2 (%)	G3/4 (%)	total	*P*
Hematological event							
White blood cell decreased	21 (32.3)	0	21 (32.3)	6 (35.2)	0	6 (35.2)	0.925
Neutropenia	18 (27.6)	10 (15.3)	28 (43.0)	3 (17.6)	8(47.0)	11 (64.7)	0.002*
Thrombocytopenia	21 (32.2)	3 (4.6)	24 (36.9)	2 (11.7)	0	2 (11.7)	0.377
Anemia	5 (7.6)	0	5 (7.6)	1 (5.8)	0	1 (5.8)	0.076
Febrile neutropenia	0	0	0	0	0	0	
Non-hematological event							
Nausea	5 (7.6)	0	5 (7.6)	10 (58.8)	2(11.7)	1 2(70.5)	< 0.001*
Anorexia	5 (7.6)	0	5 (7.6)	6 (35.2)	3(17.6)	9 (52.9)	< 0.001*
Mucositis oral	2 (3.0)	0	2 (3.0)	0	0	0	0.464
Fatigue	7 (10.7)	0	7 (10.7)	7 (41.1)	0	7 (41.1)	0.045*
AST increased	5 (7.6)	0	5 (7.6)	1 (5.8)	0	1 (5.8)	0.798
ALT increased	10 (15.3)	0	10 (15.3)	3 (17.6)	0	3 (17.6)	0.272
Creatinine increased	2 (3.0)	0	2 (3.0)	2 (11.7)	0	2 (11.7)	0.121
Constipation	14 (21.5)	0	14 (21.5)	2 (11.7)	0	2 (11.7)	0.631
Diarrhea	1 (1.5)	0	1 (1.5)	0	0	0	0.606
Pneumonitis	1 (1.5)	0	1 (1.5)	0	1(5.8)	1 (5.8)	0.128
Infection	1 (1.5)	0	1 (1.5)	0	0	0	0.606
Rash	3 (4.6)	0	3 (4.6)	0	0	0	0.665
Hypophosphatemia	1 (1.5)	0	1 (1.5)	0	0	0	0.606
Vertigo	2 (3.0)	0	2 (3.0)	1 (5.8)	0	1 (5.8)	0.583
Insomnia	1 (1.5)	0	1 (1.5)	0	0	0	0.606
Alopecia	1 (1.5)	0	1 (1.5)	0	0	0	0.606
Peripheral nerve disorder	1 (1.5)	0	1 (1.5)	0	0	0	0.606
Supraventricular tachycardia	0	1 (1.5)	1 (1.5)	0	0	0	0.606
Vasculitis	0	0	0	5 (29.4)	0	5 (29.4)	< 0.001*

*P < 0.05

**Fig. 3:**
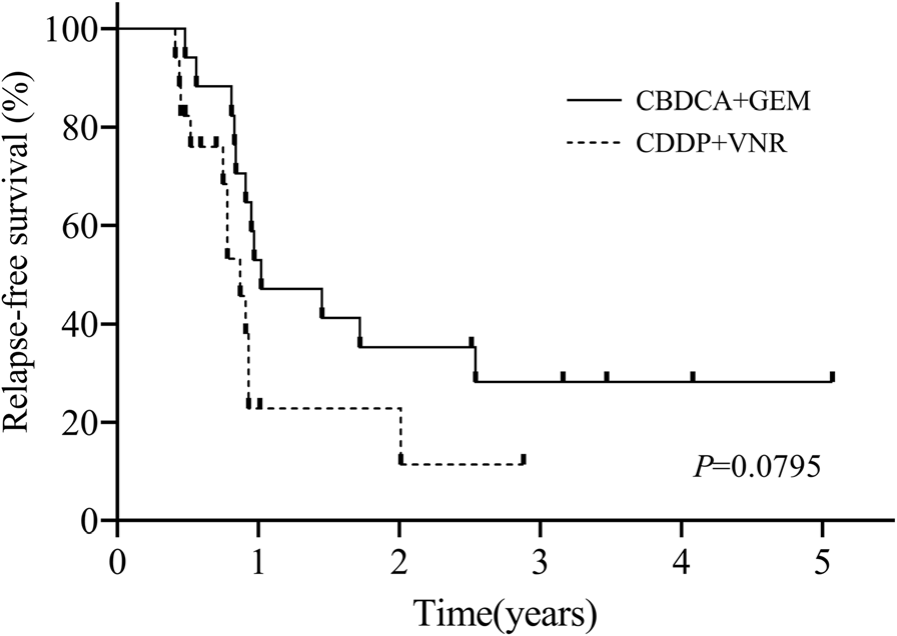
Relapse-free survival after propensity score matching

Propensity scores were generated using logistic regression based on clinically relevant variables such as age and PS, which significantly differed between the two groups, as well as pStage. Propensity score analysis yielded 17 well-matched patient pairs (Table 3). Briefly, 26.1% (17 of 65 patients) in the CBDCA + GEM group and 100.0% (17 of 17 patients) of the CDDP + VNR group were matched. Among them, 88.2% of patients in the CBDCA + GEM group completed 4 chemotherapy cycles, while 70.5% patients in the CDDP + VNR group completed 4 cycles. There were no significant differences in age, PS or pStage between the two groups after matching, and RFS also did not differ between the CBDCA + GEM and CDDP + VNR groups (*p* = 0.079) (Fig. 3).

**Table 3:** Matched patient characteristics

Matched characteristics	CBDCA + GEM (*n* = 17)	CDDP + VNR (*n* = 17)	*P*
Median age, years (range)	62 (47–70)	59 (33–70)	0.458
Gender (%)			0.243
Male	14 (82.3)	11 (64.7)	
Female	3 (17.6)	6 (35.2)	
Surgical procedure (%)			0.367
Lobectomy	17 (100.0)	16 (94.1)	
Pneumonectomy	0	1 (5.8)	
ECOG performance status (%)			1
0	17 (100)	17 (100)	
Pathological stage (%)			0.922
IIA	2 (11.7)	1 (5.8)	
IIB	3 (17.6)	4 (23.5)	
IIIA	8 (47.0)	8 (47.0)	
IIIB	4 (23.5)	4 (23.5)	
Histology (%)			0.871
Adenocarcinoma	14 (82.3)	16 (94.1)	
Squamous cell carcinoma	3 (17.6)	1 (5.8)	
Lymph nodal status (%)			0.513
N0	4 (23.5)	2 (11.7)	
N1	2 (11.7)	4 (23.5)	
N2	11 (64.7)	11 (64.7)	
Number of course			0.434
1/2/3/4	1/0/1/15	2/0/3/12	
EGFR mutation (%)			0.831
Negative/unknown	14 (82.3)	13 (76.4)	
Positive	3(17.6)	4 (23.5)	
ALK fusion (%)			0.310
Negative/unknown	17(100.0)	16(94.1)	
Positive	0	1(5.8)	

*P < 0.05

## Discussion

In the present study, we found the CBDCA + GEM regimen to be equally effective with less toxicity than the CDDP + VNR regimen currently adopted as standard adjuvant chemotherapy. CDDP-based regimens sometimes induce unacceptable toxicity and require hydration to prevent renal toxicity [23]. Consequently, CDDP-based regimens often have low patient compliance in the outpatient setting. On the other hand, the CBDCA + GEM regimen was well-tolerated and available to outpatients for short duration treatment as adjuvant chemotherapy.

Previously, CALBG 9633 (CBDCA AUC6 + PTX 200 mg/m^2^) was proposed as a CBDCA-based adjuvant chemotherapy regimen for use on an outpatient basis. However, mature results from the only RCT designed specifically for completely resected T2N0 pStage IB NSCLC patients suggested adjuvant chemotherapy with CALGB 9633 provides no significant OS advantage across the entire cohort [24]. Nonetheless, exploratory analysis demonstrated a significant improvement in both OS and disease-free survival (DFS) for patients with tumors ≥ 4 cm in diameter (HR, 0.69; 90% CI 0.48–0.99), and OS analysis indicated a 31% reduction in risk of death. In general, CDDP-based regimens had a higher response rate than CBDCA-based regimens in advanced and metastatic NSCLCs, but according to three meta-analyses there was no significant difference in the OS rate between the two platinum regimens [9, 25, 26]. Regarding toxicity, CBDCA-based regimens frequently lead to thrombocytopenia, while CDDP-based regimens frequently lead to nausea and vomiting. Given the CALBG 9633 result, the CDDP-based regimen is currently recommended as the standard adjuvant chemotherapy for patients with stage II–III NSCLC. However, the CDDP regimen is associated with a low completion rate and relatively severe toxicities in patients with resected NSCLC who have lost respiratory function due to lobectomy or pneumonectomy.

Three studies have reported on the use of CBDCA + GEM in patients with completely resected NSCLC as adjuvant chemotherapy [18–20]. Their findings indicate that the CBDCA + GEM regimen has a high chemotherapy completion rate and an acceptably low level of toxicity that includes neutropenia and thrombocytopenia. In our study, the grade 3/4 neutropenia rate was 15.3% and grade 3/4 thrombocytopenia was 4.6%, but both were easily manageable. Moreover, 88.2% of patients completed the planned 4 cycles of therapy. By contrast, the completion rate for the CDDP + VNR regimen rate was only 70.5%. The present study was retrospective study of only a small number of resected NSCLC patients, but its advantage was that of the comparison of the efficacy and tolerability of the CBDCA + GEM with the standard CDDP + VNR by propensity score matching. Especially, the 5-year RFS in squamous cell carcinoma patients treated with CBDCA + GEM was 84.2%. The CBDCA + GEM regimen might be most effective for squamous cell lung cancer. Although it may be difficult to draw a conclusion regarding the survival benefit of CBDCA + GEM from our study, we believe that CBDCA + GEM is a feasible and promising adjuvant chemotherapy option for those reasons.

Currently, the role of molecularly targeted therapies such as epidermal growth factor receptor (EGFR) tyrosine kinase inhibitors and immune checkpoint inhibitors as adjuvant therapy for early-stage NSCLC after complete surgical resection remains unclear. These included trials of the EGFR-TKIs gefitinib [27], alone and with addition of bevacizumab [11], and a trial of immunotherapy [28]. Despite the observed benefit from EGFR-TKIs for metastatic EGFR mutation-positive NSCLC, adjuvant therapy with an EGFR-TKI for 2 years did not lead to improved OS [27]. The aim of adjuvant therapy is to eradicate residual tumor cells. One of the concerns with adjuvant TKI therapy is that it can only suppress, not eliminate, the growth of residual tumor cells. Immunotherapies inhibiting programmed cell death-1 or programmed cell death-ligand 1 are now being evaluated in the adjuvant setting [12]. Based on relevant studies, including RCTs and systematic reviews [3, 5–7, 12, 27, 28], the data do not support the use of adjuvant novel chemotherapies, including TKIs, bevacizumab, or immunotherapies, either as an addition to or instead of the CDDP-based regimen. Although multiple biomarkers, including EGFR, KRAS, p53, ERCC1, b-tubulin, PARP1, p27, p16, cyclin E, and BAX, have been evaluated retrospectively, no biomarkers are currently available that are predictive of the effects of adjuvant chemotherapy. Thus, CDDP + VNR remains the standard adjuvant therapy for patients with completely resected stage II–IIIA NSCLCs. Adjuvant osimertinib showed clinically meaningful improvement in DFS in the patients with stage IB-IIIA EGFR mutation-positive NSCLC after complete tumor resection and adjuvant chemotherapy (2-year DFS rate was 89% with osimertinib vs 53% with placebo in the overall population) in the ADAURA (NCT02511106) study [29]. Further investigation will be needed to evaluate targeted agents in molecularly defined subgroups before new agents can be recommended in the adjuvant setting [12].

The TREAT trial was a randomized phase II trial in patients with resected early stage NSCLC, which tested the hypothesis that a CDDP + pemetrexed (PEM) protocol with reduced toxicity would improve adjuvant chemotherapy drug delivery, compliance, and survival [30]. Although adjuvant chemotherapy with CDDP + PEM is safe and less toxic than the standard CDDP + VNR, OS was not influenced in the treatment arm whether the histologic diagnosis was squamous cell carcinoma or adenocarcinoma. A randomized phase III trial of CDDP + PEM vs. CDDP + VNR as adjuvant chemotherapy for non-squamous pStage II-IIIA NSCLC is ongoing. Although the survival results have yet to be published, it is anticipated that a better cure rate with a new regimen with lower toxicity, such as CDDP + PEM, will be achieved in the near future. However, we believe that CBDCA + GEM as adjuvant chemotherapy may leads to avoid the ineffective use of CDDP/CBDCA + PEM protocol, which have the most effective benefit with less toxicity as cytotoxic chemotherapy for the patients with recurrence/advanced adenocarcinoma, if each overall survival is not influenced.

This study has several limitations. First, it was retrospective in nature and included only a small number of patients followed up for a short period in the CDDP + VNR group, which may have led to suboptimal results. Second, the OS data in the CDDP + VNR group are premature. However, RFS after adjuvant chemotherapy in the present study is a valid surrogate endpoint for OS that is not confounded by crossover of subsequent chemotherapy for recurrence. Third, physicians decided on whether or not to administer adjuvant chemotherapy, and this selection bias could lead to insignificant results of treatment. Fourth, we could not analyze some factors in the propensity score matching because there were not a sufficient number of analytic cases. Fifth, we did not analyze patients receiving adjuvant UFT. The Japan Lung Cancer Research Group (JLCRG) study of adjuvant UFT is a well-known RCT, in which patients with pStage I lung adenocarcinoma were randomly assigned to 2 years of adjuvant chemotherapy with oral UFT or observation [31]. Adjuvant UFT improved survival among patients with completely resected pT2N0 adenocarcinoma. Although UFT is a commonly used oral chemotherapy in Japan, we excluded it from our analysis because it is not available for lung cancer everywhere in the world.

In summary, we found adjuvant chemotherapy with CBDCA + GEM to be effective for disease control and to be well tolerated by patients with completely resected NSCLC. The CBDCA regimen represents a potential treatment option suitable for use on an outpatient basis in clinical practice.

## Data Availability

The datasets used and/or analyzed during the current study are available from the corresponding author on reasonable request.
